# Does Presentation of Late-Life Generalized Anxiety Disorder Change with Advancing Age?

**DOI:** 10.1093/geroni/igaf122.3573

**Published:** 2025-12-31

**Authors:** Yusuf Lahlou, Philippe Landreville, Patrick Gosselin, Sébastien Grenier, Pierre-Hugues Carmichael

**Affiliations:** Université Laval, Québec, Quebec, Canada; Université Laval, Québec, Quebec, Canada; Université de Sherbrooke, Sherbrooke, Quebec, Canada; Université de Montréal, Montréal, Quebec, Canada; Centre d’Excellence sur le Vieillissement de Québec, Québec, Quebec, Canada

## Abstract

Generalized anxiety disorder (GAD) is one of the most common anxiety disorders in older adults. Given the aging population, it is important to determine if there are age-related variations in the presentation of GAD in older adults. The present study examined the relationship between age and the clinical presentation of GAD in people aged 60 to 87 years (N = 109, M = 68.2). A structured diagnostic interview was conducted and participants completed measures of GAD severity, tendency to worry, behavioral manifestations of GAD, depression, and disability. Most of the analyses performed did not show a significant relationship between age and different indices of GAD presentation. A comparison of the youngest (≤67 years) and oldest (>67 years) participants, based on the median age of the sample, showed that the second group had significantly higher rates of comorbid anxiety disorders (60.8% vs 39.7%; χ² = 4.85, p = .03) and death-related worries (54.9% vs 34.5%; χ² = 4.44, p = .04). Conversely, the youngest group reported more work or school-related worries (60.5% vs 25.0%; χ² = 9.15, p = .002). Overall, the results show that the clinical presentation of GAD does not vary much according to age among older adults. However, the observed differences suggest that clinicians should take into account the presence of comorbid anxiety disorders and the sources of worries according to the age of their client.

